# Agricultural land-use change in a Mexican oligotrophic desert depletes ecosystem stability

**DOI:** 10.7717/peerj.2365

**Published:** 2016-08-18

**Authors:** Natali Hernández-Becerra, Yunuen Tapia-Torres, Ofelia Beltrán-Paz, Jazmín Blaz, Valeria Souza, Felipe García-Oliva

**Affiliations:** 1 Laboratorio de biogeoquímica de suelos, Instituto de Investigaciones en Ecosistemas y Sustentabilidad, UNAM, Morelia, Michoacán, Mexico; 2 ENES Unidad Morelia, Universidad Nacional Autónoma de México, Morelia, Michoacán, Mexico; 3 Instituto de Ecología, Universidad Nacional Autónoma de México, Mexico

**Keywords:** Alfalfa, Resilience, Bacteria community, Soil nutrients, Microbial activity, *Medicago sativa* L.

## Abstract

**Background:**

Global demand for food has led to increased land-use change, particularly in dry land ecosystems, which has caused several environmental problems due to the soil degradation. In the Cuatro Cienegas Basin (CCB), alfalfa production irrigated by flooding impacts strongly on the soil.

**Methods:**

In order to analyze the effect of such agricultural land-use change on soil nutrient dynamics and soil bacterial community composition, this work examined an agricultural gradient within the CCB which was comprised of a native desert grassland, a plot currently cultivated with alfalfa and a former agricultural field that had been abandoned for over 30 years. For each site, we analyzed C, N and P dynamic fractions, the activity of the enzyme phosphatase and the bacterial composition obtained using 16S rRNA clone libraries.

**Results:**

The results showed that the cultivated site presented a greater availability of water and dissolved organic carbon, these conditions promoted mineralization processes mediated by heterotrophic microorganisms, while the abandoned land was limited by water and dissolved organic nitrogen. The low amount of dissolved organic matter promoted nitrification, which is mediated by autotrophic microorganisms. The microbial N immobilization process and specific phosphatase activity were both favored in the native grassland. As expected, differences in bacterial taxonomical composition were observed among sites. The abandoned site exhibited similar compositions than native grassland, while the cultivated site differed.

**Discussion:**

The results suggest that the transformation of native grassland into agricultural land induces drastic changes in soil nutrient dynamics as well as in the bacterial community. However, with the absence of agricultural practices, some of the soil characteristics analyzed slowly recovers their natural state.

## Introduction

Rising global food demand due to population growth has caused an increase in rates of land-use change to agricultural production in dry ecosystems (Lepers et al., 2005; Reynolds et al., 2007). This has led to several environmental problems, including deforestation, habitat fragmentation, biodiversity reduction, changes to global biogeochemical cycles, water and soil contamination and degradation (Reynolds et al., 2007; Rey-Benayas & Bullock, 2012). In these perturbed dry lands, the main drivers of desertification are soil nutrient losses caused mainly by erosion, soil salinization and the reduction of soil water retention capacity through the deterioration of soil physical properties (D’Odorico et al., 2013). This soil degradation reduces agricultural productivity and the fields are eventually abandoned.

The main characteristics of intensive agriculture that affect soil properties is the reduction of organic matter inputs, soil tillage, fertilization and irrigation (McLauchlan, 2006). It has been reported that soil organic matter (SOM) is reduced by 16–77%, as a consequence of agriculture (Murty et al., 2002). This is mainly through the decrease in organic matter inputs and the increase in soil organic decomposition because of increased tillage and soil temperatures (Trasar-Cepeda et al., 2008; Beheshti, Raiesi & Golchin, 2012). The practice of tillage disrupts the physical properties of the soil, affecting soil water and nutrient dynamics (Six, Elliott & Paustian, 1999; Zeleke et al., 2004; Bronick & Lal, 2005). Fertilization with nitrogen, mainly in the form of ammonium, promotes faster nitrification and the release of H^+^ ions into the soil solution, thus lowering soil pH (Moore, Klose & Tabatabai, 2000) and the continuous irrigation increases the leaching of salts through the soil profile (Raiesi, 2004). However, when agricultural fields are abandoned, some salts accumulate in the topsoil, promoting salinization, a process that is favored in desert ecosystems (Rietz & Haynes, 2003; Pan et al., 2012). Furthermore, plant succession is slower in desert ecosystems than in wet tropical ecosystems; for example, recovery of vegetation requires at least 40 years in the former, while in the latter it can be achieved in less than 10 years (Lesschen et al., 2008; Wang et al., 2011).

Agriculture also has an effect on the composition of the soil microbial community. For instance, some changes in microbial composition have been reported as a result of agricultural land-use in tropical (Waldrop, Balser & Firestone, 2000) as well as desert (Ding et al., 2013) and Mediterranean ecosystems (Garcia-Orenes et al., 2013). However, the effect on soil microbial diversity is unclear; some studies have described increases in biodiversity (Jangid et al., 2008) while others have reported decreases (Lupwayi, Rice & Clayton, 1998). Chaudhry et al. (2012) found higher soil microbial diversity in agricultural fields managed with organic rather than chemical fertilization. These authors found that the composition of the bacterial community in the organically fertilized soil was dominated by the phyla Proteobacteria, Bacteroidetes and Gemmatimonadetes, while the groups Actinobacteria and Acidobacteria were predominant in the chemically fertilized soil. The dominant phyla in the organically fertilized soil have been associated with high nutrient availability, whereas the Acidobacteria have been related to nutrient-poor soils (Fierer, Bradford & Jackson, 2007). The effect of long-term agricultural management on soil microbial communities is similarly unclear; in some studies, even after 9 years of abandonment, the soil microbial composition remains similar to that of the cultivated soil (Buckley & Schmidt, 2001). However, an agricultural field abandoned for over 45 years presented a soil microbial community that was similar to one in soil with native vegetation cover (Buckle & Schmidt, 2003). These results demonstrate the need for further study in order to understand the effect of succession of agriculture management upon the composition of the soil microbial community.

The worldwide area of degraded agriculture fields was estimated to be 12,400,000 km^2^ in 2007 (Rey-Benayas & Bullock, 2012), of which 20% corresponded to dry ecosystems (Lepers et al., 2005; Reynolds et al., 2007). In Mexico, around 121 km^2^ and 45 km^2^ of grassland were converted to agriculture and abandoned lands, respectively, between 2005 and 2010 (Colditz, Llamas & Ressl, 2014). For this reason, evaluation of the capacity for soil restoration in the cultivated fields of dry lands is a priority for crop production and ecosystem conservation. This capacity can be evaluated in the context of ecosystem stability, which has two main components: resistance and resilience (Pimm, 1984). The former is the capacity of the ecosystem to face a disturbance without undergoing structural changes, while the latter reflects the time required for the ecosystem to return to its pre-disturbance condition (Pimm, 1984). Orwin & Wardle (2004) proposed indices for evaluating these two attributes of soil stability, which are accurate for providing a relative quantitative measurement when comparing soil conditions under perturbation. The quantitative measure of soil stability allows evaluation of the magnitude of soil degradation and its capability for restoration.

In the Cuatro Cienegas basin (CCB) in Mexico, alfalfa (*Medicago sativa* L.) production with gravity irrigation involves flooding the fields with oasis water that is channeled through open canals for hundreds of km. This practice unequivocally threatens the sustainability of the CCB wetland and degrades the soil and vegetation. In order to analyze the effect of such agricultural land-use on the soil nutrient dynamics (C, N and P) and composition of the soil bacterial community, we examined an agricultural gradient within the CCB composed of three sites with the same soil type but under contrasting management: a native desert grassland, a plot with an alfalfa crop and a former agricultural field that had been abandoned for over 30 years. We predicted that the alfalfa production disrupts the mechanisms of soil nutrient transformation and strongly affects the composition of the soil bacteria. To test these hypotheses, we analyzed C, N and P dynamic fractions and used this data to calculate the homeostasis of the microbial community. The enzymatic activity of alkaline phosphatase was also quantified and bacterial composition was determined through the use of 16S rRNA clone libraries.

## Material and Methods

### Site description

This study was carried out in the Cuatro Cienegas basin (CCB; 26°50′N and 102°8′W) at 740 masl, in the Chihuahuan desert in Mexico. The climate is seasonally arid with an average annual temperature of 21 °C and annual precipitation of 252 mm (http://smn.cna.gob.mx/). Jurassic-era gypsum is the dominant parent material on the western side of the basin, while Jurassic-era limestone dominates on the eastern side (McKee, Jones & Long, 1990). According to the WRB classification (2007), the predominant soils are Gypsisol and Calcisol on the western and eastern sides of the basin, respectively. The soil within the CCB is characterized by low P concentrations (ranging between 70–200 µg g^−1^). These values are lower than the *P* values of other soils within the Chihuahuan desert (500–1,000 µg g^−1^; Tapia-Torres & Garcia-Oliva, 2013). The main vegetation types are halophyte-grassland dominated by *Sporobolus airoides* (Poaceae) and desert scrub dominated by species from the Euphorbiaceae and Zygophyllaceae families (Perroni, Garcia-Oliva & Souza, 2014). Agricultural activity in the CCB began in the early decades of the 20th century but has increased in the last 30 years and it mainly consists of the production of alfalfa for cattle fodder. Alfalfa (*Medicago sativa* L.) is grown by flooding the fields and introducing large quantities of fertilizer. In some years, sorghum (*Sorghum* spp.) is cultivated, but the alfalfa cultivation dominates the agricultural surface (INEGI, 2011). However, these fields must eventually be abandoned due to degradation of the soil, mainly through salinization.

### Field sampling

Sampling sites were located on the eastern side of the CCB. An agricultural gradient was established comprising three sites of shared soil type (Calcisol) but contrasting management was all located in flat areas: native desert grassland, a plot cultivated with alfalfa and a former agricultural field that had been abandoned for over 30 years. The native desert grassland was in the Pozas Azules reserve (26°49′30″N and 102°1′27″W) where *Sporobolus airoides* is the dominant plant species (Tapia-Torres et al., 2015a). The cultivated alfalfa field was located in the Cuatro Cienegas ejido (26°58′47″N and 102°02′13″W) and covered an area of 2.7 ha with high fertilizer inputs and irrigation by flooding every month. The plot was fertilized with monoammonium phosphate (11-52-00) dissolved in the water for irrigation. The water for irrigation had a pH value of 8.5 with a high electrical conductivity (150 mS m^−1^). This alfalfa plot has been under cultivation for 20 consecutive years and the alfalfa is harvested every month. Finally, the abandoned field was also in the Cuatro Cienegas ejido (26°58′57″N and 102°01′8″W) and presented minimum plant cover (less than 30% of the area). Oscar Sánchez Liceaga, Héctor Castillo González, the personnel of APFF Cuatro Cienegas (CONANP) and the people in charge of Rancho Pozas Azules (PRONATURA) gave us the permission to collect soil samples on their respective properties. At each site, a 100 × 50 m plot was delimited and then divided into 10 sections at a distance of 10 m apart. A random sampling transect was then established in each section, with topsoil samples taken to a depth of 15 cm at ten sampling points (every five meters) in September 2011; these samples were then mixed to form one composite sample. In total, 10 such composite samples were taken in each plot. Soil for biogeochemical and enzymatic activity analysis was stored in black plastic bags and refrigerated at 4 °C. In order to characterize the bacterial community at each site, 100 g of composite samples were immediately stored in liquid nitrogen until subsequent DNA extraction.

### Laboratory analyses

#### Soil nutrient and enzymatic analyses

Soil pH was measured in deionized water (1:2 w:v) using a digital pH meter (Corning) and soil electrical conductivity was measured by conductivity meter (Hannan Instruments Inc., Houston, USA). A subsample (100 g) was oven-dried at 75 °C to constant weight for soil moisture determination using the gravimetric method in order to adjust for water content when expressing nutrient concentration on the basis of dry soil mass. All C forms analyzed in all samples were determined in a total carbon analyzer (UIC model CM5012, Chicago, USA), while the N and P forms analyzed were determined colorimetrically in a Bran-Luebbe Auto analyzer 3 (Norderstedt, Germany). Prior to the total soil nutrient analyses, soil samples were dried and ground with a pestle and mortar. Total carbon (TC) and inorganic carbon (IC) were determined by combustion and coulometric detection (Huffman, 1977). Total organic carbon (OC) was calculated as the difference between TC and IC. For total N (TN) and total P (TP) determination, samples were acid digested with H_2_SO_4_, H_2_O_2_, K_2_SO_4_ and CuSO_4_ at 360 °C. Soil N was determined by the macro-Kjeldahl method (Bremmer, 1996), while P was determined by the molybdate colorimetric method following ascorbic acid reduction (Murphy & Riley, 1962).

Available, dissolved and microbial nutrient forms were extracted from field moist soil samples. Available inorganic N (NH${}_{4}^{+}$ and NO${}_{3}^{-}$) was extracted from 10 g of fresh soil subsamples with 2M KCl, followed by filtration through a Whatman No. 1 paper filter (Robertson et al., 1999) and determined colorimetrically by the phenol-hypochlorite method. Available (inorganic) and labile (organic) P was determined by extraction with 0.5M NaHCO_3_ at pH 8.5 according to Hedley sequential P fractionation (Tiessen & Moir, 1993) and quantified as described above for orthophosphate.

Dissolved nutrients were extracted with deionized water after shaking for 45 min and filtering through a Millipore 0. 42 00B5m filter (Jones & Willett, 2006). Prior to acid digestion, one aliquot of the filtrate was used to determine dissolved ammonium (DNH${}_{4}^{+}$) and inorganic P (IP) in deionized water extract. Total dissolved nitrogen (TDN) was digested using the macro-Kjeldahl method. Total dissolved P (TDP) was also acid digested and determined by colorimetry. Total dissolved carbon (TDC) was measured with an Auto Analyzer of carbon (TOC CM 5012) module for liquids (UIC-COULOMETRICS). Inorganic dissolved carbon (IDC) was determined in an acidification module CM5130. Dissolved organic carbon (DOC), dissolved organic nitrogen (DON) and dissolved organic phosphorous (DOP) were calculated as the difference between total dissolved forms and inorganic dissolved forms.

Microbial C (C_mic_), N (N_mic_) and P (P_mic_) concentrations were determined by the chloroform fumigation extraction method (Vance, Brookes & Jenkinson, 1987). Fumigated and non-fumigated samples were incubated for 24 h at 25 °C and constant moisture. Microbial C was extracted from fumigated and non-fumigated samples with 0.5 M K_2_SO_4_ and filtered through Whatman No. 42 filters (Brookes et al., 1985). The concentration of C was measured in each extract as total and inorganic C concentration by the method described before. Microbial C was calculated by subtracting the extracted carbon in non-fumigated samples from that of fumigated samples and dividing the result by a K_*EC*_ value (the extractable part of microbial biomass C) of 0.45 (Joergensen, 1996). Microbial N was extracted with the same procedure used for C_mic_, but the extract was filtered through Whatman No. 1 paper. The filtrate was acid digested and determined as TN by Macro-Kjeldahl method (Brookes et al., 1985). Microbial N was calculated as for C_mic_, but divided by a K_*EN*_ value (the extractable part of microbial biomass N after fumigation) of 0.54 (Joergensen & Mueller, 1996). Microbial P was extracted using NaCO_3_ 0.5M at pH 8.5, after which the fumigation-extraction technique involving chloroform was performed (Cole et al., 1978). Microbial P was calculated as for C_mic_ and N_mic_ and converted using a K_*P*_ value (the extractable part of microbial biomass P after fumigation) of 0.4 (Lathja et al., 1999). Microbial P was determined colorimetrically by the molybdate-ascorbic acid method using an Evolution 201 Thermo Scientific Inc. spectrophotometer (Murphy & Riley, 1962). Finally, C_mic_, N_mic_ and P_mic_ values were normalized on a dry soil basis.

Because P is considered the most limited soil nutrient in the east-side of the CCB (Tapia-Torres et al., 2015), alkaline phosphatase activity was analyzed colorimetrically using *ρ*-nitrophenol (*ρ*NP) substrates, according to Tabatabai & Bremner (1969) and Eivazi & Tabatabai (1977). For this analysis, 2 g of fresh soil and 30 ml of modified universal buffer (MUB) at pH 9 were used for the exoenzyme extraction. Three replicates and one control (sample without substrate) were prepared per sample. Three substrate controls (substrate without sample) were also included per assay. We centrifuged the tubes after the incubation period and then 750 µl of supernatant was diluted in 2 ml of deionized water and absorbance of *ρ*-nitrophenol (*ρ*NP) measured at 410 nm on an Evolution 201 Thermo Scientific Inc, spectrophotometer. Exoenzyme activities were expressed as micromoles of *ρ*NP formed per gram dry weight of soil per hour (µmol *ρ*NP [g SDW]^−1^ h^−1^). This value was standardized by C_mic_ concentration for expression as a specific enzyme activity (µmol *ρ*NP [mg C_mic_]^−1^ h^−1^).

#### Molecular analyses

Total DNA was extracted using the hydroxyapatite spin-column method (Purdy et al., 1996). DNA molecular weight and quality were confirmed using agarose gel electrophoresis. The 16S rRNA gene was amplified from each sample using a polymerase chain reaction (PCR) with the universal primers F27 (5′*AGAGTTTGATCMTGGCTCAG*3′) and R1492 (5′*GGTTACCTTGTTACGACTT*3′). Three independent PCRs were performed for each sample. The PCR reactions were 50 µl in volume and contained 2µl of DNA, 1 µl PCR buffer 1× 0.5 mM MgCl_2_, 0.2 mM dNTP mixture, 0.2 mM of each primer, 1 unit of platinum Taq DNA Polymerase High Fidelity (Invitrogen), 5% DMSO and 0.05 mg of BSA. The PCR was performed in a thermal cycler (MJ Research, Watertown, MA) under the following cycling program: initial denaturation step at 94 °C for 5 min, then 30 cycles at 94 °C for 1 min, 52 °C for 1 min,and 72 °C for 1 min 20 sec, with a final extension step at 72 °C for 30 min and storage at 4 °C. The three reactions were pooled and purified in a 1% agarose gel using the QIAquick gel extraction kit (Qiagen). The purified fragment was cloned into the vector PCR 2.1 and transformed into Escherichia coli following the manufacturer’s instructions (Invitrogen). Only plasmids containing inserts were isolated for sequencing with the Montage Plasmid Miniprepkit (Millipore). The insertion within the plasmids was sequenced with the Sanger method using the vector-based primer 27F.

### Data analysis

#### Stoichiometric homeostasis

The degree of community-level microbial C:N and C:P homeostasis (*H*′) by soil microorganisms was calculated with the formula proposed by Sterner & Elser (2002):

(1) \begin{eqnarray*}{H}^{{^{\prime}}}=1/m.\end{eqnarray*}

In Eq. (1), *m* is the slope of log_*e*_ C:N_*R*_ (Carbon and Nitrogen in the resources) versus log_*e*_ C:N_*B*_ (Carbon and Nitrogen in the microbial biomass) or slope of log_*e*_ C:P_*R*_ (Carbon and Phosphorus in the resources) versus log_*e*_ C:P_*B*_ (Carbon and Phosphorus in the microbial biomass) scatterplot. *H*′ ≫ 1 represents strong stoichiometric homeostasis, while *H*′ ≈ 1 represents weak or no homeostasis (Sterner & Elser, 2002).

#### Resistance and resilience index

Nutrient concentration and enzymatic activity data were both analyzed for resistance and resilience using the indices proposed by Orwin and Warlde (2004). The grassland site was considered as the control (C_0_), the cultivated site as the disturbance (P_0_) and the abandoned plot was used for measuring resilience 30 years after the cessation of agriculture management (P_*x*_). Resistance (RS) was calculated as follows:

(2) \begin{eqnarray*}\mathrm{RS}=1-((2{|}{\mathrm{D}}_{0}{|})/({\mathrm{C}}_{0}+{|}{\mathrm{D}}_{0}{|})).\end{eqnarray*}

In Eq. (2), C_0_ represents the control soil and D_0_ is the difference between C_0_ and the disturbed plot (P_0_). In addition, resilience (RL) was calculated as follows:

(3) \begin{eqnarray*}\mathrm{RL}=((2{|}{\mathrm{D}}_{0}{|})/({|}{\mathrm{D}}_{0}{|}+{|}{\mathrm{D}}_{X}{|}))-1(3).\end{eqnarray*}

In Eq. (3), D_*X*_ is the difference between C_0_ and P_*x*_. Both indexes are bounded by −1 and +1, if the value is −1 means less resistance or resilience, while the +1 value means maximal resistance or resilience.

#### Bioinformatics analysis

Sequencing quality evaluation as well as cloning vector removal were performed using the sorftware PHRED (Ewing & Green, 1998). For processing and classification of the sequence data, the open source software package Mothur (v 1.15.0; Schloss et al., 2009) was used. Sequences were screened for potential chimeric reads using Chimera.slayer (Haas et al., 2011) and the linked SILVA template database. High-quality sequences were compared against the SILVA database in order to obtain their taxonomic rank. A pairwise distance matrix was calculated across the non-redundant sequences, and reads were clustered into operational taxonomic units (OTUs) at 3% distance, using the furthest neighbor method (Schloss & Handelsman, 2005). In addition, the Simpson and Shannon (H) indices, Chao species richness estimator and rarefaction curves were estimated.

#### Statistical analysis

One-way ANOVA was used to identify differences in nutrient concentrations and enzymatic activity between the sites of the agricultural gradient (grassland, cultivated field and abandoned field). Log-transformations were applied where the data deviated from normality. When ANOVA indicated a significant site effect, mean comparisons were performed with Tukey’s multiple comparisons test (Von Ende, 1993).

Pearson correlations were used to explore relationships among soil parameters. Principal Components Analysis (PCA) was conducted in order to group soil samples with active nutrients forms (dissolved, available and microbial) and enzymatic activity. Similarly, Canonical Analysis was conducted with soil nutrients (available, dissolved organic and pH) as the independent variables and nutrients within microbial biomass and phosphatase activity as dependent variables. All analyses were performed using R software 2.10.1 (R Development Core Team, 2009).

## Results

### Soil nutrients

#### Soil nutrients

The abandoned and cultivated plots had the highest and the lowest soil pH and soil electrical conductivity, respectively (*P* < 0.0001 and *P* = 0.0002 for pH and electrical conductivity, respectively; Table 1). Total organic C, N and P concentrations differed among management gradient plots. Total organic C was almost two times greater in the cultivated plot than in the other two plots (*P* < 0.0001; Table 1), whereas the cultivated and grassland plots presented the highest and the lowest N and P concentrations, respectively (*P* < 0.001 and *P* < 0.0001 for N and P, respectively; Table 1). As a consequence, the highest C:P and N:P ratios were in the grassland plot (*P* < 0.0001 for both C:P and N:P), while the C:N ratio did not differ among plots (Table 1).The cultivated plot presented higher DOC and DOP than the other two plots (*P* < 0.0001 and *P* < 0.001 for DOC and DOP, respectively), but DON presented no differences among plots (Table 1). Similarly, the cultivated plot presented a greater concentration of ammonium than the other two plots (*P* < 0.0001), but the highest values of nitrate and available P were in the abandoned and the grassland plots, respectively (*P* < 0.0001 for both NO_3_ and available P; Table 1).

**Table 1:** Means (standard error) of available, dissolved, microbial forms of C, N and P and Specific phophonatase activity (SPA) of soil from an agricultural gradient at Cuatro Ciénegas Basin. Values immediately followed by a different letter indicate that the means are significantly different (*P* ≤ 0.05) among agricultural gradient plots.

	Grassland	Cultivated plot	Abandoned plot
pH	8.5 (0.03)^B^	7.9 (0.04)^C^	8.8 (0.04)^A^
EC (mS m^−1^)	8.7 (0.6)^B^	3.4 (0.1)^C^	15.6 (3.0)^A^
TOC (mg g^−1^)	5.97 (0.71)^B^	21.50 (1.17)^A^	9.54 (1.49)^B^
TN (mg g^−1^)	0.63 (0.06)^C^	2.61 (0.07)^A^	1.13 (0.05)^B^
TP (mg g^−1^)	0.094 (0.01)^C^	0.768 (0.04)^A^	0.53 (0.02)^B^
C:N	9.3 (0.3)	8.3 (0.6)	8.3 (1.2)
C:P	64 (5)^A^	29 (2)^B^	18 (3)^C^
N:P	6.9 (0.5)^A^	3.5 (0.2)^B^	2.1 (0.1)^C^
DOC (µg g^−1^)	9 (2)^C^	116 (9)^A^	39 (7)^B^
DON (µg g^−1^)	7.7(0.8)	6.6 (0.2)	13.6 (3.5)
DOP (µg g^−1^)	1.1 (0.3)^B^	14.6 (0.2)^A^	2.1 (0.8)^B^
NH${}_{4}^{+}$ (µg g^−1^)	1.64 (0.08)^B^	3.51 (0.40)^A^	1.55 (0.13)^B^
NO${}_{3}^{-}$ (µg g^−1^)	0^C^	4.91 (0.41)^B^	18.16 (1.30)^A^
HPO${}_{4}^{-}$ (µg g^−1^)	0.096 (0.015)^A^	0.010 (0.002)^B^	0.004 (0.001)^B^
C_mic_ (µg g^−1^)	108 (12)^B^	451 (68)^A^	145 (29)^B^
N_mic_ (µg g^−1^)	14 (1.3)^B^	95 (23.6)^A^	4 (1.0)^C^
P_mic_ (µg g^−1^)	1.95 (0.41)^B^	5.88 (1.21)^A^	3.20 (0.48)^*AB*^
C_mic_:N_mic_	8.1 (0.9)	9.00 (2.3)	23 (6.9)
C_mic_:P_mic_	42 (9)	99 (17)	56 (13)
N_mic_:P_mic_	5.3 (1.1)^A^	33.2 (16.4)^B^	1.7 (0.3)^A^
SPA (µm mgC${}_{\mathrm{mic}}^{-1}$ h^−1^)	1.50 (0.44)^A^	0.57 (0.08)^B^	0.46 (0.27)^B^

**Notes.**

EC Electrical conductivity TOC totalorganic Carbon TN total Nitrogen TP total Phophorus DOC dissolved organic Carbon DON dissolved organic nitrogen DOP dissolved organic phosphorus NH${}_{4}^{+}$ ammonium NO${}_{3}^{-}$ nitrate HPO${}_{4}^{-}$ orthophosphate C_mic_ microbial carbon N_mic_ microbial nitrogen P_mic_ microbialphosphorus SPA specific phosphatase activity

#### Nutrients within microbial biomass

The cultivated plot had higher C and N concentrations within the microbial biomass (*P* < 0.0001 for both C_mic_ and N_mic_), but did not differ from the abandoned plot in terms of microbial P (Table 1). However, the grassland plot had higher N_mic_ concentration than the abandoned plot and, consequently, the C:N and C:P ratios of the microbial biomass did not differ among plots, but the N:P ratio was highest in the cultivated plot (*P* = 0.05).

Using the equation for C:N and C:P homeostasis (*H*′), the soil microbial community did present a strong elemental homeostasis for phosphorus acquisition in the three sites (*H*′ = 6.25, 9.35 and 12.9 respectively for cultivated, grassland and abandoned plots). For nitrogen acquisition, however, the microbial community of the cultivated soil presented a weak homeostasis (*H*′ = 0.63), while the grassland (3.23) and abandoned plot (5.29) presented higher homeostasis.

#### Enzymatic activity

**Figure 1:**
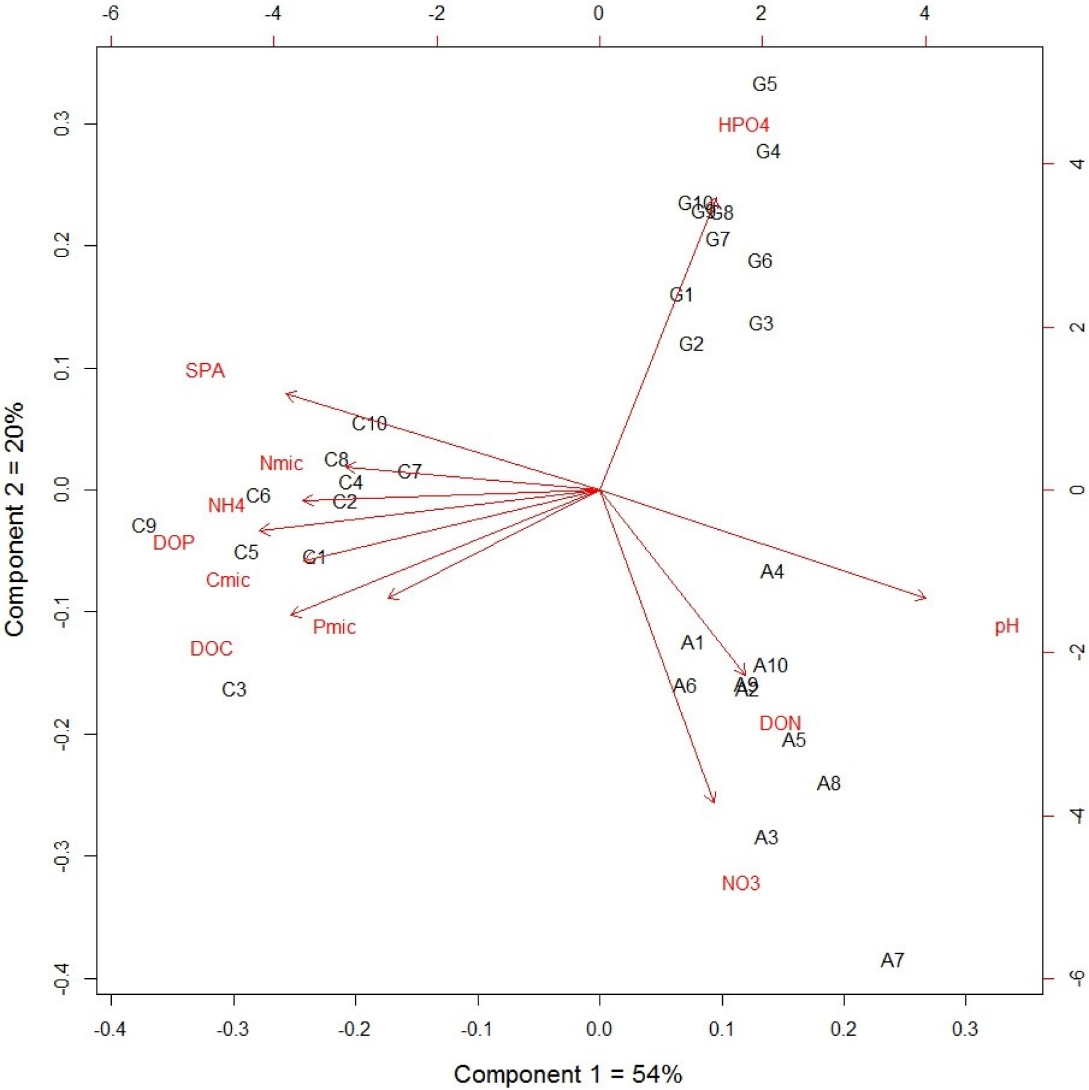
Principal component analysis of dynamic nutrient forms from an agricultural gradient at Cuatro Cienegas Basin.

The grassland soil had higher specific phosphatase activity than the other two managed plots (*P* < 0.0001; Table 1). The DOC correlated positively with DOP, ammonium, nutrients within microbial biomass and phosphanatase activity, while nitrate correlated negatively with available P and phosphanatase activity (Table 2). The first two principal components explained 74% of the total variance, in which 54% was explained by the first component. In the first component, the cultivated plot differed statistically to the other two non-cultivated plots, while all three plots were significantly different in the second component (Fig. 1). These results suggest that the difference between the cultivated plot and the other two plots explained 54% of the total variance in the soil nutrient dynamic. The dynamic forms of soil nutrients strongly correlated with nutrients within microbial biomass and phosphatase activity as determined by canonical analysis (Canonical *R* = 0.98, *P* < 0.0001). The eigenvalue of root 1 was 0.95 and pH and POD had the highest canonical weight in root 1.

**Table 2:** Pearson correlation coefficients for available nutrients and nutrients within microbial biomass in soil from agricultural gradient at Cuatro Cienegas Basin.

	pH	DOC	DON	DOP	NH${}_{4}^{\ast }$	NO${}_{3}^{-}$	HPO${}_{4}^{-}$	C_mic_	N_mic_	P_mic_	SPA
pH	1										
DOC	−0.70^*^	1									
DON	0.46^*^	−0.12	1								
DOP	−0.85^*^	0.88^*^	−0.37^*^	1							
NH${}_{4}^{+}$	−0.68^*^	0.65^*^	−0.23	0.72^*^	1						
NO${}_{3}^{-}$	0.59^*^	−0.01	0.46^*^	−0.19	−0.21	1					
HPO${}_{4}^{-}$	0.09	−0.51^*^	−0.17	−0.44	−0.27	−0.61^*^	1				
C_mic_	−0.68^*^	0.79^*^	−0.24	0.74^*^	0.70^*^	−0.09	−0.32	1			
N_mic_	−0.70^*^	0.52^*^	−0.22	0.66^*^	0.67^*^	−0.18	−0.20	0.44^*^	1		
P_mic_	−0.41^*^	0.68^*^	−0.21	0.57^*^	0.39^*^	−0.01	−0.30	0.62^*^	0.15	1	
SPA	−0.88^*^	0.65^*^	−0.40^*^	0.84^*^	−0.76^*^	−0.52^*^	−0.11	0.64^*^	0.62^*^	0.30	1

**Notes.**

* Means significant correlation at *P* ≤ 0.05.

DOC dissolved organic Carbon DON dissolved organic nitrogen DOP dissolved organic phosphorus NH4+ ammonium NO${}_{3}^{-}$ nitrate HPO${}_{4}^{-}$ orthophosphate C_mic_ microbial carbon N_mic_ microbial nitrogen P_mic_ microbial phosphorus SPA specific phosphatase activity

### Soil resistance and resilience

In general, the soil variables analyzed showed low resistance to agricultural management, since the majority of the resistance values were negative or close to zero, with the exception of pH and DON (Table 3). Similarly, the soil variables also had low resilience, because none of the values was close to 1 (Table 3), which means that these soil variables were dissimilar to the grassland soil. However, the C and N concentrations within the microbial biomass, DOC and DOP were closer in value to 1 (above 0.5), suggesting that these soil variables were more resilient than the other soil variables analyzed (Table 3), although these values were insufficient to achieve recovery of these soil variables after 30 years.

**Table 3:** Mean values (±standard error) of the resistance and resilience values of nutrient parameters from an agricultural gradient at Cuatro Cienegas Basin.

Variable	Resistance	Resilience
pH	0.88 (±0.01)	0.20 (±0.12)
DOC	−0.81 (±0.06)	0.61 (±0.06)
DON	0.54 (±0.08)	−0.28 (±0.18)
DOP	−0.84 (±0.04)	0.81 (±0.06)
NH${}_{4}^{+}$	0.04 (±0.15)	0.42 (±0.16)
NO${}_{3}^{-}$	−1.00 (±0.00)	−0.57 (±0.03)
HPO${}_{4}^{+}$	0.08 (±0.02)	−0.04 (±0.02)
C_mic_	−0.43 (±0.09)	0.56 (±0.13)
N_mic_	−0.45 (±0.16)	0.56 (±0.15)
P_mic_	−0.28 (±0.17)	0.37 (±0.13)
SPA	−0.06 (0.10)	0.25 (±0.12)

**Notes.**

TITLE DOC dissolved organic Carbon DON dissolved organic nitrogen DOP dissolved organic phosphorus NH4^+^ ammonium NO3^−^ nitrate HPO4^−^ orthophosphate C_mic_ microbial carbon N_mic_ microbial nitrogen P_mic_ microbial phosphorus SPA specific phosphatase activity

### Soil bacteria composition

#### Composition of bacterial communities

**Figure 2:**
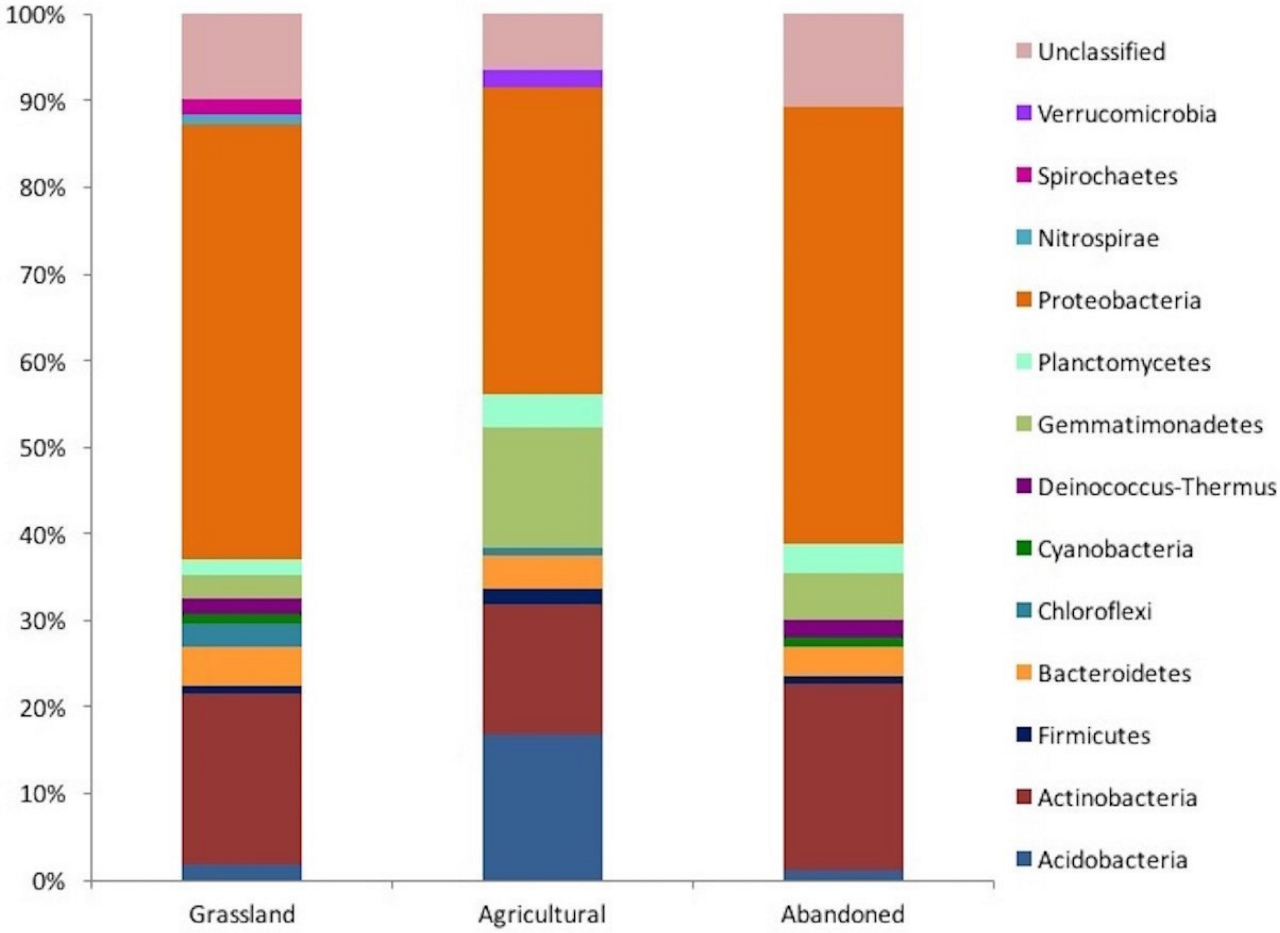
Taxonomic distribution of the 16 rRNA gene sequences obtained from clone libraries of an agricultural gradient at Cuatro Cienegas Basin.

A total of 111 sequences were obtained for the grassland, 107 sequences for the cultivated plot and 93 sequences for the abandoned site. In the grassland, we obtained a clone library with 111 sequences, while the cultivated plot had 107 sequences and the abandoned plot had 93. In the grassland, the sequences were distributed among 12 phyla and 19 classes, while the cultivated plot sequences comprised 9 phyla and 14 classes, and those of the abandoned plot comprised 9 phyla and 12 classes. These results suggest that the bacterial community of the grassland soil was distributed in higher phyla than was the case in the other two managed plots. For example, Protobacteria was the more abundant bacteria phylum in the three plots, accounting for 50% of the results in the grassland and the abandoned plot, but representing only 35% in the cultivated plot (Fig. 2). Similarly, Actinobacteria was the second most dominant phylumin both the grassland and abandoned plot (20% and 21%, respectively), but only represented 15% in the cultivated plot. The two most important phototrophic phyla (Chloroflexi and Cyanobacteria) were not found in the cultivated plot, but Cyanobacteria was found in both the grassland soil and abandoned plot (Fig. 2).

**Figure 3:**
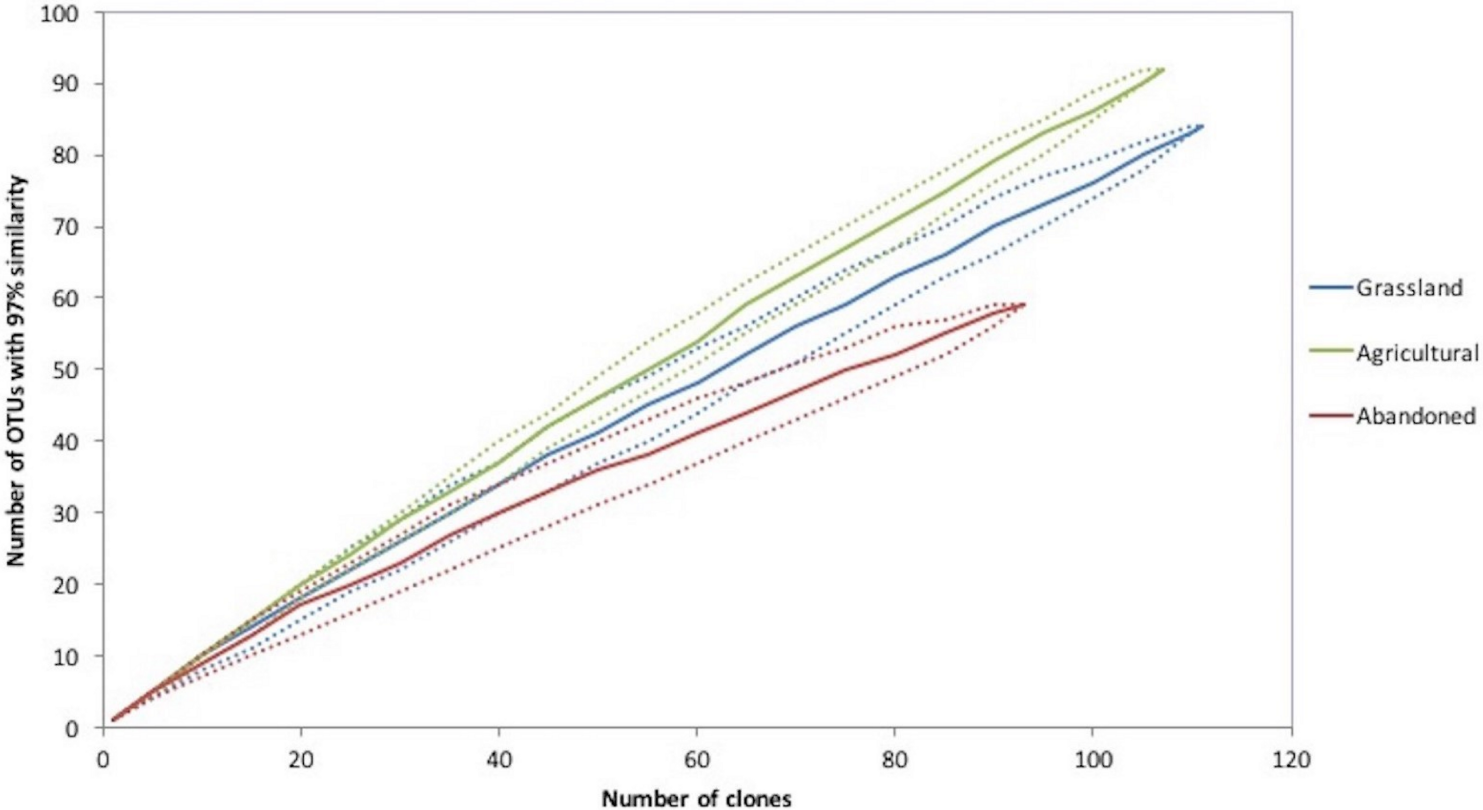
Rarefaction curves of an agricultural gradient at Cuatro Cienegas Basin. OTUs were determined at 97% sequence identity.

#### Diversity of bacterial communities

Rarefaction curve analysis showed that the cultivated plot had the richest bacterial community, followed by the abandoned plot and finally the grassland soil (Fig. 3). In addition, the cultivated plot had the highest expected OTUs by the Chao analyses (659), while the abandoned plot had the lowest expected value of OTUs (179). The latter plot also had the lowest values of Simpson and Shannon indices (*D* = 0.025 and *H* = 3.8, respectively), suggesting that the bacterial community of the abandoned plot was dominated by fewer OTUs in comparison with the bacterial communities in the cultivated plot and the grassland soil (*D* = 0.04, *H* = 4.4 and *D* = 0.013, *H* = 4.2; respectively).

From the total of 307 sequences obtained for all sites, 223 OTUs were recognized at 97% of similitude. The cultivated plot again had the highest number of OTUs (92), followed by grassland (84 OTUs) and finally the abandoned plot with the lowest number of OTUs (59). The three sites shared four OTUs corresponding to the Proteobacteria (Rhizobiales, Pseudomonadales, Burkholderiales and Xanthomonadales). The abandoned plot shared two OTUs with the other sites, but there were no OTUs shared between the grassland and the cultivated plot. Finally, the grassland soil and abandoned plot presented higher similitude between them relative to the cultivated plot, using the 16S rRNA community composition at 97% similarity based on the Bray-Curtis algorithm.

## Discussion

### Soil nutrient dynamics

In the Cuatro Cienegas basin (CCB), alfalfa production by flooding the fields threatens the wetlands sustainability and contributes to the degradation of soil and vegetation system. The results showed that the cultivated plot presented a lower soil pH than the other two sites, which could be associated with the fertilization and continuous irrigation, it has been reported in other agriculture sites (Moore, Klose & Tabatabai, 2000; Raiesi, 2004). Soil N fertilization mainly with ammonium, as it is applied to the site of the present study, promotes nitrification by releasing H^+^ ions into the soil solution (Moore, Klose & Tabatabai, 2000), while continuous irrigation increases the leaching of salt through the soil profile (Raiesi, 2004). However, the cultivated plot presented higher concentrations of total C, N and P than the other two plots. These increases are caused by fertilization and by the particular crop under cultivation, with the latter mainly affecting the SOC concentration. Perennial legumes, such as alfalfa, promote higher SOC accumulation in comparison with the annual crops since they feature high root biomass production and require low soil tillage (Franzluebbers, 2009; Sainju & Lenssen, 2011; Bell et al., 2012; Yang et al., 2013). Furthermore, the alfalfa plot had a greater availability of dissolved organic carbon (DOC), which could be explained by higher organic matter input and soil water availability. These conditions promoted depolymerization of organic molecules and mineralization of organic nutrients mediated by the activity of heterotrophic microorganisms (Wardle, 1992; Vineela et al., 2008). Associated with this higher activity of heterotrophic microorganisms, organic N is mainly released as NH${}_{4}^{+}$ and then immobilized within microbial biomass, as suggested by the NH${}_{4}^{+}$ and N_mic_ values of the cultivated plot. All of these results suggest that the cultivated plot presented higher soil nutrient transformations, mainly of N, promoted by the availability of water and nutrient fertilization, and thus the soil nutrient dynamics of this plot differ from the plots without management, as suggested by the results of the PCA. In contrast, the low amount of soil organic matter in the native grassland is consequence of low availability of soil water in the east-side of CCB (Tapia-Torres et al., 2015a). The low water availability reduces plant productivity and in consequence there is a lower input of organic matter input to the soil, as Tapia-Torres et al. (2015b) reported for soils under desert scrub within CCB. Consequently, the activity of microbial populations is constrained by low availability of organic carbon (Wardle, 1992).

The nutrients within microbial biomass and phosphatase activity are strongly affected by the dynamics of soil nutrients as Canonical Analysis confirmed. While the cultivated plot presented higher nutrient concentrations within microbial biomass than the other two plots, microbial C:N and C:P did not differ among plots. These results suggest that the soil microbial community had different strategies for nutrient acquisition in order to equilibrate nutrient stoichiometry (Sterner & Elser, 2002). The soil microbial communities of the plots showed elemental homeostasis, with the exception of the cultivated soil, in which N acquisition showed weak homeostasis, probably in response to the constant fertilization with ammonium. Tapia-Torres et al. (2015a) also reported a strong N and P homeostasis for two native grasslands within the CCB. These results suggest that soil microbial communities adopt different strategies for nutrient acquisition, including the production of eco-enzymes which clave the organic molecules for microbial assimilation (Waring, Weintraub & Sinsabaugh, 2014). Phosphatase is the main eco-enzyme that mineralizes organic P molecules (Tabatabai & Bremner, 1969). In our study site, the native grassland had higher specific phosphatase activity, indicating that members of the soil microbial community in this plot invest more in production of this enzyme than in growth, which suggests that this microbial community is co-limited by C and P as reported before for the same study site by Tapia-Torres et al. (2015a). Moreover, the microbial C:N:P ratio of the cultivated plot (99:33:1) is wider than that proposed by Cleveland & Liptzin (2007) for different terrestrial ecosystems (60:7:1), while the non-managed plots are closer to this ratio (42:5:1 and 56:2:1 for the natural grassland and abandoned plot, respectively). These results suggest that the agricultural management strongly disrupts soil microbial activity and its homeostasis.

As expected, the sites under no current management were limited by water and DOC. At the abandoned site, these conditions promoted the nitrification process, which is mediated by autotrophic microorganisms that can use NH${}_{4}^{+}$ as their energy source (Hart et al., 1994; Chen & Stark, 2000). The microbial N immobilization process was favored in the native grassland; this process promotes N conservation within the ecosystem, as previously reported for native grassland in the CCB (Tapia-Torres et al., 2015b).

### Soil bacteria composition

The agricultural land-use change affected the soil bacteria composition. Agricultural management increased the numbers of OTUs and diversity indices associated with higher availability of soil water and energy for microbial activity. Such increases due to agriculture activity have been reported for other desert sites (Köberl et al., 2011; Wang et al., 2012). However, the abandoned plot had lower OTUs and diversity indexes in comparison with the other two plots, probably associated with more stressful soil conditions (i.e., higher salinity, lower water and nutrient availability) as reported by Keshri, Mody & Jha (2013) for desert soils.

The two dominant phyla from the three plots analyzed were Proteobacteria and Actinobacteria, which are both very common in agricultural (Buckle & Schmidt, 2003; Chaudhry et al., 2012) and desert (Chanal et al., 2006; López-Lozano et al., 2012) soils. However, their relative proportion differed among plots, especially in the case of the cultivated plot. Moreover, the two most important phototrophic phyla (Chloroflexi and Cyanobacteria) were not found in the cultivated plot, where N input and soil disruption selected against their presence. As expected, Cyanobacteria were present in both the grassland soil and the abandoned plot, forming a desert crust (Li et al., 2012). In contrast, the Acidobacteria were more abundant in the cultivated plot (*ca*. 18%), while in the non-cultivated plot had decreased to 2%. This phylum is associated with pH neutral or acid soils, such as the soils of the cultivated plot. The results suggest that agricultural management has a strong effect on soil bacterial composition, because the agricultural plot shared lower OTUs (only 4) with the plot under no management. Furthermore, according to the Bray-Curtis algorithm, the grassland soil and the abandoned plot had a higher similitude between them relative to the cultivated plot. For example, in both the native grassland and the abandoned plots, some extremophile OTUs were present, e.g., that associated with the phylum Deinococcus-Thermus, which is adapted to stressful soil conditions such as salinity, high temperatures, aridity, etc., (Nienow, 2009); however, these OTUs were not presented in the cultivated plot. These results suggest that some OTUs recover after abandonment of agricultural management, although the soil bacteria community is not yet similar to that in the native grassland even after more than 30 years since abandonment. One study has reported similar soil bacteria in native vegetation and sites abandoned for over 45 years in agro-ecosystems of Michigan State (Buckle & Schmidt, 2003).

In soil microbial communities, microfungi are an important and diverse component of microbial diversity, representing a large proportion of microbial diversity in soils (Fierer, Bradford & Jackson, 2007). These microorganisms play an immense role in regulating energy and nutrient fluxes through natural ecosystems, via their involvement in soil development, decomposition and uptake of nutrients by plants (Dighton, 1997) mainly phosphate uptake. Future studies should be aimed at understanding the role of microfungi in soil nutrient cycling in this ecosystem. However, tagging of ITS markers for soil fungi in CCB have been challenging, so there is still further research needed in this field.

### Soil resistance and resilience

All of the variables evaluated presented low resistance and resilience, suggesting that the native grassland soil may be very vulnerable to agricultural transformation. The resilience of soil is determined by its intrinsic characteristics, as well as by prevailing climatic conditions (Blanco-Canqui & Lal, 2010). For instance, soil with high organic matter content is more resilient, since organic compounds represent important reservoirs of energy and nutrients for both the soil microbial community and plants (Bronick & Lal, 2005). In addition, ecosystems in humid climates are also more resilient than arid ecosystems because they are not constrained by water availability. For example, the wet tropical ecosystem requires less than 10 years for recovery of its vegetal community following perturbation, while the desert ecosystem requires at least 40 years (Lesschen et al., 2008; Wang et al., 2011). Our results suggest that the native grassland presents slow recovery and this characteristic is critical for the design of alternative agricultural management, as well as appropriate strategies for soil reclamation. This is important because the rate of soil degradation is faster than that of soil restoration, which acts to increase the area of degraded lands in these arid ecosystems.

The design of soil restoration practices is critical for CCB, because the ecosystems within CCB are very vulnerable to the disruption of nutrient dynamics, and the native species have low competition capacity against invasive species under higher availability of resources (Souza et al., 2006). This situation is critical for the soils of CCB, because they contain a high diversity of native species that can face up the scarcity of nutrients, mainly P (Tapia-Torres et al., 2016). The organic agriculture with low pesticide inputs and the use of native microbial strains with different capabilities to use, transform and recycle the soil nutrients (i.e., phosphorus solubilizing bacteria) could be the best solution for agriculture in this particular and highly diverse important ecosystem. These agricultural practices not only will allow the maintenance of soil microbial biodiversity but also will contribute to the soil conservation. Therefore, ensuring long-term availability and accessibility to healthy soil, mainly for food security is a global challenge.

## Conclusions

Our results suggest that land-use change transforming native grassland into agricultural land induces drastic modifications in the soil nutrient dynamics as well as in the bacterial community. However, with the suspension of agricultural practices, some soil characteristics tend to slowly recover their natural state.

##  Supplemental Information

10.7717/peerj.2365/supp-1 Data S1 Raw data

## Additional Information and Declarations

### Field Study Permissions

The following information was supplied relating to field study approvals (i.e., approving body and any reference numbers): Oscar Sánchez Liceaga, Héctor Castillo González, the personnel of APFF Cuatro Cienegas (CONANP) and the people in charge of Rancho Pozas Azules (PRONATURA) gave us permission to collect soil samples on their respective properties. The owners gave us oral permission to collect soil samples.

### Data Availability

The following information was supplied regarding data availability: The raw data has been supplied as Data S1.

## References

[ref-1] Beheshti A, Raiesi F, Golchin A (2012). Soil properties, C fractions and their dynamics in land use conversion from native forest to croplands in a northern Iran. Agriculture, Ecosystem and Environment.

[ref-2] Bell LW, Sparling B, Tenuta M, Entz MH (2012). Soil profile carbon and nutrient stocks under long-term conventional and organic crop and alfalfa-crop rotations and re-established grassl. Agriculture Ecosystem & Environment.

[ref-3] Blanco-Canqui H, Lal R (2010). Soil resilience and conservation. Principles of soil conservation and management.

[ref-4] Bremmer JM, Spark DL, Page AL, Summer ME, Tabatabai MA, Helmke PA (1996). Nitrogen-Total. Methods of soil analyses part 3: chemical analyses, soil science.

[ref-5] Bronick CJ, Lal R (2005). Soil structure and management: a review. Geoderma.

[ref-6] Brookes P, Landman A, Pruden G, Jenkinson D (1985). Chloroform fumigation and the release of soil nitrogen: a rapid direct extraction method to measure microbial biomass nitrogen in soil. Soil Biology and Biochemistry.

[ref-7] Buckle DH, Schmidt TM (2003). Diversity and dynamics of microbial communities in soils from agro-ecosystems. Environmental Microbiology.

[ref-8] Buckley DH, Schmidt TM (2001). The structure of microbial communities in soil and the lasting impact of cultivation. Microbial Ecology.

[ref-9] Chanal A, Chapon V, Benzerara K, Barakat M, Christen R, Achouak W, Barras F, Heulin T (2006). The desert of Tataouine: an extreme environment that hosts a wide diversity of microorganisms and radio tolerant bacteria. Environmental Microbiology.

[ref-10] Chaudhry V, Rehman A, Mishra A, Chauhan PS, Nautiyal CC (2012). Changes in bacterial community structure of agriculture land due to long-term organic and chemical amendments. Microbial Ecology.

[ref-11] Chen J, Stark JM (2000). Plant species effects carbon and nitrogen cycling in a sagebrush-crested wheatgrass soil. Soil Biology Biochemistry.

[ref-12] Cleveland CC, Liptzin D (2007). C:N:P stiochiometry in soil: is there a “Redfield ratio” for the microbial biomass?. Biogeochemistry.

[ref-13] Colditz RR, Llamas RM, Ressl RA (2014). Detecting change areas in Mexico between 2005 and 2010 using 250 m MODIS images. IEEE Journal on Selected Topics in Applied Earth Observation and Remote Sensing.

[ref-14] Cole CV, Elliott ET, Hunt HW, Coleman DC (1978). Trophic interactions in soils as they affect energy and nutrient dynamics. V. Phosphorus transformations. Microbial Ecology.

[ref-15] Dighton J (1997). Nutrient cycling by saprotrophic fungi in terrestrial habitats. The Mycota.

[ref-16] Ding G, Piceno YM, Heuer H, Weinert N, Dohrmann AB, Carrillo A, Andersen GL, Castellanos T, Tebbe CC, Smalla K (2013). Changes of soil bacteria diversity as a consequence of agriculture land use in a semi-arid ecosystem. PLoS ONE.

[ref-17] D’odorico P, Bhattachan A, Davis KF, Ravi S, Runyan CW (2013). Global desertification: drivers and feedback. Advances in Water Resources.

[ref-18] Eivazi F, Tabatabai MA (1977). Phosphatases in soils. Soil Biology and Biochemistry.

[ref-19] Ewing B, Green P (1998). Base-calling of automated sequencer traces using phred. II. Error probabilities. Genome Research.

[ref-20] Fierer N, Bradford MA, Jackson RB (2007). Toward an ecological classification of soil bacteria. Ecology.

[ref-21] Franzluebbers AJ (2009). Achieving soil organic carbon sequestration with conservation agricultural systems in the southeastern United States. Soil Science Society of American Journal.

[ref-22] Garcia-Orenes F, Morugán-Coronado A, Zornoza R, Scow K (2013). Changes in soil microbial community structure influenced by agricultural management practices in a Mediterranean Agro-Ecosystem. PLoS ONE.

[ref-23] Haas BJ, Gevers D, Earl AM, Feldgarden M, Ward DV, Giannoukos G, Ciulla D, Tabba D, Highlander SK, Sodergren E, Methé B, Desantis TZ, human microbiomes Consortium T, Petrosino JF, Knight R, Birren BW (2011). Chimeric 16S rRNA sequence formation and detection in Sanger and 454-pyrosequenced PCR amplicons. Genome Research.

[ref-24] Hart S, Nason GE, Myrlod D, Perry DA (1994). Dynamic of gross nitrogen transformations in an old-growth forest: the carbon connection. Ecology.

[ref-25] Huffman EN (1977). Performance of a new automatic carbon dioxide coulometer. Microchemical Journal.

[ref-26] INEGI, Instituto Nacional de Estadstica y Geografia II (2011). Anuario Estadistico de Coahuila de Zaragoza.

[ref-27] Jangid K, Williams MA, Franzluebbers AJ, Sanderlin JS, Reeves JH, Jenkins MB, Endale DM, Coleman DC, Whitman WB (2008). Relative impacts of land-use, management intensity and fertilization upon soil microbial community structure in agricultural systems. Soil Biology and Biochemistry.

[ref-28] Joergensen RG (1996). The fumigation-extraction method to estimate soil microbial biomass: Calibration of the K_*EC*_ value. Soil Biology and Biochemistry.

[ref-29] Joergensen RG, Mueller T (1996). The fumigation-extraction method to estimate soil microbial biomass: calibration of de K_*EN*_ value. Soil Biology and Biochemistry.

[ref-30] Jones DL, Willett VB (2006). Experimental evaluation of methods to quantify dissolved organic nitrogen (DON) and dissolved organic carbon (DOC) in soil. Soil Biology and Biochemistry.

[ref-31] Keshri J, Mody K, Jha B (2013). Bacterial community structure in a semi-arid haloalkaline soil using culture independent method. Geomicrobiology Journal.

[ref-32] Lathja K, Driscoll CT, Jarrell WM, Elliott ET, Robertson GP, Coleman DC, Bledsoe CS, Sollins P (1999). Soil phosphorus: characterization and total element analysis. Standard soil methods for long-term ecological research.

[ref-33] Lepers E, Lambin EF, Janetos AC, Fries RD, Archad F, Ramankutty N, Scholes RJ (2005). A synthesis of rapid land-cover change information for the 1981–2000 period. BioScience.

[ref-34] Lesschen JP, Cammeraat LH, Kooijman AM, Wesemael B (2008). Development of spatial heterogeneity in vegetation and soil properties after land abandonment in a semi-arid ecosystem. Journal of Arid Environment.

[ref-35] Li XR, Zhang P, Su YG, Jia RL (2012). Carbon fixation by biological soil crusts following revegetation of sand dunes in arid desert regions of China: a four-year field study. Catena.

[ref-36] López-Lozano NE, Eguiarte LE, Bonilla-Rosso G, Garcia-Oliva F, Martinez-Piedragil C, Rooks C, Souza V (2012). Bacteria communities and nitrogen cycle in the gypsum soil in CuatroCienegas Basin, Coahuila: a Mars analogue. Astrobiology.

[ref-37] Lupwayi NZ, Rice WA, Clayton GW (1998). Soil microbial diversity and community structure under wheat as influenced by tillage and crop rotation. Soil Biology and Biochemistry.

[ref-38] McKee JW, Jones NW, Long LE (1990). Stratigraphy and provenance of strata along the San Marcos fault, central Coahuila, Mexico. Geological Society of America Bulletin.

[ref-39] McLauchlan KK (2006). The nature and longevity of agriculture impacts on soil carbon and nutrients: a review. Ecosystems.

[ref-40] Moore JM, Klose S, Tabatabai MA (2000). Soil microbial biomass carbon and nitrogen as affected by cropping systems. Biology and Fertility of Soils.

[ref-41] Murphy J, Riley JP (1962). A modified single solution method for the determination of phosphate in natural waters. Analytica Chimica Acta.

[ref-42] Murty D, Kirschbaum MUF, McMurtrie RE, McGilvray H (2002). Does conversion of forest to agricultural land change soil carbon and nitrogen? A review of the literature. Global Change Biology.

[ref-43] Nienow J, Schaechter M (2009). Extremophiles: dry environments (including cryptoendoliths). Encyclopedia of microbiology.

[ref-44] Orwin KH, Wardle DA (2004). New indices for quantifying the resistence and resilience of soil biota to exogenous disturbances. Soil Biology and Biochemistry.

[ref-45] Pan C, Liu C, Zhao H, Wang Y (2012). Changes of soil physical-chemical properties and enzyme activities in relation to grassland salinization. European Journal of Soil Biology.

[ref-46] Perroni Y, Garcia-Oliva F, Souza V (2014). Plant species identity and soil P forms in an oligotrophic grassland–desert scrub system. Journal of Arid Environments.

[ref-47] Pimm SL (1984). The complexity and stability of ecosystems. Nature.

[ref-48] Purdy KJ, Embley TM, Takii S, Nedwell DB (1996). Rapid extraction of DNA and rRNA from sediments by a novel hydroxyapatite spin-column method. Applied and Environmental Microbiology.

[ref-49] R Development Core Team (2009).

[ref-50] Raiesi F (2004). Soil properties and N application effects on microbial activities in two winter wheat cropping systems. Biology and Fertility of Soils.

[ref-51] Rey-Benayas JM, Bullock JM (2012). Restoration of biodiversity and ecosystem services on agricultural land. Ecosystems.

[ref-52] Reynolds JF, Smith D, Lambin EF, Turner BL, Mortimore M, Batterbury S, Downing TE, Dowlatabadi H, Fernández RJ, Herrick JE, Huber-Sannwald E, Juang H, Leemans R, Lynam T, Maestre FT, Ayarza M, Walker B (2007). Global desertification: building a science for dryland development. Science.

[ref-53] Rietz D, Haynes R (2003). Effects of irrigation-induced salinity and sodicity on soil microbial activity. Soil Biology and Biochemistry.

[ref-54] Robertson PG, Coleman DC, Bledsoe CS, Sollins P (1999). Standard soil methods for long-term ecological research (LTER).

[ref-55] Sainju UM, Lenssen AW (2011). Dryland soil carbon under alfalfa and durum-forage cropping sequences. Soil Tillage Research.

[ref-56] Schloss PD, Handelsman J (2005). Introducing DOTUR, a computer program for defining operational taxonomic units and estimating species richness. Applied Environmental Microbiology.

[ref-57] Schloss PD, Westcott SL, Ryabin T, Hall JR, Hartmann M, Hollister EB, Lesniewski RA, Oakley BB, Parks DH, Robinson CJ, Sahl JW, Stres B, Thallinger GG, Van Horn DJ, Weber CF (2009). Introducing mothur: open- source, platform-independent, community-supported software for describing and comparing microbial communities. Applied Environmental. Microbiology.

[ref-58] Six J, Elliott ET, Paustian K (1999). Aggregate and soil organic matter dynamics under conventional and no-tillage systems. Soil Science Society of America Journal.

[ref-59] Souza V, Esponosa_Asuar L, Escalante AE, Eguiarte LE, Farmer J, Forney L, Lloret L, Rodríguez-Martínez JM, Soberon X, Dirzo R, Elser JJ (2006). An endangered oasis of aquatic microbial biodiversity in the Chihuahuan Desert. Proceedings of the National Academy of Sciences of the United States of America.

[ref-60] Sterner RW, Elser JJ (2002). Ecological stoichiometry: the biology of elements from molecules to the biosphere.

[ref-61] Tabatabai MA, Bremner JM (1969). Use of p-Nitrophenyl phosphate for assay of soil phosphatase activity. Soil Biology and Biochemistry.

[ref-62] Tapia-Torres Y, Elser JJ, Souza V, García-Oliva F (2015a). Ecoenzymatic stoichiometry at the extremes: How microbes cope an ultra-oligotrophic desert soil. Soil Biology and Biochemistry.

[ref-63] Tapia-Torres Y, Garcia-Oliva F (2013). La disponibilidad del fósforo es producto de la actividad bacteriana en el suelo en ecosistemas oligotróficos: una revisión crítica. Terra-Latinoamericana.

[ref-64] Tapia-Torres Y, López-Lozano NE, Souza V, García-Oliva F (2015b). Vegetation-soil system controls soil mechanisms for nitrogen transformations in an oligotrophic Mexican desert. Journal of Arid Environments.

[ref-65] Tapia-Torres Y, Rodríguez-Torres MD, Islas A, Elser J, Souza V (2016). How to live with phosphorus scarcity in soil and sediment: lessons from bacteria. Applied and Environmental Microbiology.

[ref-66] Tiessen H, Moir JO, Carter MR, Gregorich EG (1993). Characterization of available P by sequential extraction. Soil sampling and methods of analysis.

[ref-67] Trasar-Cepeda C, Leirós MC, Seoane S, Gil-Sotres F (2008). Biochemical properties of soils under crop rotation. Applied Soil Ecology.

[ref-68] Vance ED, Brookes AC, Jenkinson DS (1987). An extraction method for measuring soil microbial biomass C. Soil Biology and Biochemistry.

[ref-69] Vineela C, Wani SP, Srinivasarao C, Padmaja B, Vittal KPR (2008). Microbial properties of soils as affected by cropping and nutrient management practices in several long-term manorial experiments in the semi-arid tropics of India. Applied Soil Ecology.

[ref-70] Von Ende CN, Scheiner SM, Gurevitch J (1993). Repeated measures analysis: growth and other time-dependent measures. Design and analysis of ecological experiments.

[ref-71] Waldrop MP, Balser TC, Firestone MK (2000). Linking microbial community composition to function in a tropical soil. Soil Biology and Biochemistry.

[ref-72] Wang B, Liu G, Xue S, Zhu B (2011). Changes in soil physico-chemical and microbiological properties during natural succession on abandoned farmland in the Loess Plateau. Environmental Earth Science.

[ref-73] Wang B, Zhang C, Liu J, Zeng X, Li F, Wu Y, Lin X, Xiong ZQ, Xu J, Jia ZJ (2012). Microbial community changes along a land-use gradient of desert soil origin. Pedosphere.

[ref-74] Wardle DA (1992). A comparative assessment of factors which influence microbial biomass carbon and nitrogen levels in soil. Biological Reviews.

[ref-75] Waring BG, Weintraub SR, Sinsabaugh RL (2014). Ecoenzymatic stoichiometry of microbial nutrient acquisition in tropical soils. Biogeochemistry.

[ref-76] Yang R, Su Y, Gan Y, Du M, Wang M (2013). Field-scale spatial distribution characteristics of soil nutrients in a newly reclaimed sandy cropland in the Hexi Corridor of Northwest China. Environmental Earth Science.

[ref-77] Zeleke TB, Grevers MCJ, Si BC, Mermut AR, Beyene S (2004). Effect of residue incorporation on physical properties of the surface soil in the South Central Rift Basin of Etiopia. Soil Tillage Research.

